# What’s Really Wrong with Fining Crimes? On the Hard Treatment of Criminal Monetary Fines

**DOI:** 10.1007/s11572-021-09623-3

**Published:** 2021-11-25

**Authors:** Ivó Coca-Vila

**Affiliations:** Present Address: Criminal Law Department, Max Planck Institute for the Study of Crime, Security and Law, Freiburg, Germany

**Keywords:** Monetary fine, Expressive theories, Punishment, Censure, Hard treatment, Obstruction of punishment enforcement

## Abstract

Among the advocates of expressive theories of punishment, there is a strong consensus that monetary fines cannot convey the message of censure that is required to punish serious crimes or crimes against the person (e.g., rape). Money is considered an inappropriate symbol to express condemnation. In this article, I argue that this sentiment is correct, although not for the reasons suggested by advocates of expressivism. The monetary day-fine should not be understood as a simple deprivation of money, but as a punishment that reduces the offender’s capacity to consume for a certain period of time. Conceived in this manner, I argue that it is perfectly suitable to convey censure. However, the practical impossibility of ensuring that the person who pays the fine is the same person who has been convicted of the offense seriously undermines the acceptability of the monetary fine as an instrument of censure. Minimizing the risk of the fine’s hard treatment being transferred to third parties is a necessary condition for the monetary fine to be considered a viable alternative to lengthy prison sentences.

## Introduction

Although contemporary legal scholars and philosophers of punishment notoriously focus on imprisonment when discussing the concept and justification of criminal punishment,^1^ the fact remains that monetary fines are the most widely used form of criminal sanction in the majority of Western penal systems. In Germany, for example, roughly 80% of all criminal sanctions imposed on individuals are monetary fines.^2^ While the specifics of each system differ, in Europe, the monetary fine is commonly imposed as a single punishment (i.e., not as a supplement to imprisonment) that is determined by a two-stage decision process (day-fine system).^3^ First, the number of day-fine units to which the offender is sentenced is determined with regard to the gravity of the offense. Second, the quantum of every day-fine unit is assessed by setting it explicitly in relation to the offender’s income or wealth. The amount of the fine is then calculated by multiplying these numbers together. Even though a one-off payment of the total fine amount is common practice, the original design of the day-fine system deemed payment by installment as a necessary feature of the fine.^4^

The benefits of monetary day-fines over imprisonment are many.^5^ On the one hand, day-fines can be highly individualized in proportional response to the offense and to the offender’s financial situation, insofar as the number of day-fine units is assessed with regard to the seriousness of the offense and the quantum of the fine unit with regard to the offender’s personal income or wealth. On the other hand, it is a less desocializing and less stigmatizing form of punishment. It involves no physical coercion and is not as intrusive as imprisonment, as it does not require constant and absolute state supervision. It is largely reversible in the event of injustice and it permits flexible and individual enforcement (i.e., payment in installments) that take into consideration the changing personal circumstances of the convicted person. Furthermore, it is significantly more efficient for the state that imposes it. It is relatively easy to administer and –unlike custodial punishments– it actually brings in money to the public coffers.

In view of the benefits of the day-fine system, and taking into consideration economic developments in Europe since the Second World War (where the large majority of citizens, even those who commit crimes, have a regular, measurable, and legal income flow),^6^ it is not surprising that the fine has ended up being the main mode of punishment – in quantitative terms – in European criminal systems and has been presented as the ideal penal measure. Its progressive expansion at the expense of imprisonment from the 1970s onwards has been welcomed by legal scholars of widely divergent orientations as a sign of the humanization of criminal law.^7^ While initially only short prison sentences were replaced by monetary fines, this method of punishment is now also used for crimes of intermediate gravity. For a long time, it seemed that it was only a matter of time before continental European states would entirely give up custodial sentences in favor of criminal monetary fines and community service.^8^

However, this process of expanding the monetary fine as a viable punishment seems to have reached its end. According to a widely held opinion among continental and Anglo-American legal philosophers who embrace the so-called expressive theory of punishment,^9^ the monetary fine can be an appropriate response to minor or intermediate offenses but never to grave crimes or, at least, never to certain types of serious crimes (e.g., sexual crimes). Unlike punishments affecting very personal goods —such as life, physical integrity, freedom, or honor— a monetary fine, insofar as it takes the form of a simple monetary obligation, would be inappropriate to convey the message of condemnation or censure that punishing non-minor crimes requires. Fining really does not involve a statement by the polity that a given form of conduct is disgraceful. Dan Kahan, probably the most prominent critic of the criminal monetary fine as a replacement for imprisonment, concludes that both kinds of punishment are incommensurable: “because imprisonment and its rivals don’t *say* the same thing, no politically acceptable exchange rate can be constructed between them for purposes of criminal punishment.”^10^ According to this view, despite the many advantages of the day-fine, it cannot completely replace imprisonment. To the extent that non-punitive measures or other less severe punishments such as community service orders are not generally considered to be real alternatives to middle or long-term custodial sentences, imprisonment remains the only suitable realistic response to serious criminal offenses that still respects the dignity of the criminal offender.^11^

Here, I argue that the proposition that we cannot punish serious crimes through monetary fines is true. However this is not due to the reasons alleged by advocates of expressivism. The uniqueness claim —that only imprisonment can express the degree of censure or condemnation called for by serious offenses— is mistaken, even assuming an expressive or communicative foundation of punishment. Contrary to what it may seem, this does not mean that we are able to properly punish core criminal law offenses with monetary fines. This article, then, has two major ambitions. The first is to show that the thesis of the punishment incommensurability or non-interchangeability between imprisonment and fines rests on an incorrect understanding of the monetary fine as punishment and, in particular, of its hard treatment dimension. I argue that a proper understanding of the monetary fine makes this kind of punishment in principle a suitable response to all sorts of crimes in the framework of contemporary Western criminal law systems. The second ambition of this article is to defend the thesis that the main obstacle for the progressive replacement of imprisonment by a monetary fine is not its inadequacy to communicate censure, but the practical problem of ensuring that the hard treatment of the monetary fine falls on the convicted and not on third parties who have nothing to do with the offense committed. This problem —largely unnoticed or underestimated by legal and philosophical scholars— is a challenging one for defenders of the monetary fine as an alternative punishment. It is, however, not intractable.

To these ends, I begin in Sect. 2 by offering some background by outlining an expressive conception of punishment and drafting the arguments commonly raised against the possibility of resorting to monetary fines to punish serious crimes. In Sect. 3, I analyze the hard treatment aspect of monetary fines and argue that they are indeed a suitable response to serious offenses. In Sect. 4, I show that the real problem of resorting to fines to punish any kind of crime is the high risk that they will not be “absorbed” by the person who has been declared culpable. I then outline some legal ways to minimize this risk. In particular, I advocate for the criminalization of the payment of another person’s fine as an offense of obstruction of punishment enforcement. In Sect. 5, I conclude the analysis.

## What Money Can’t Say

It is currently widely accepted that the standard case of legal punishment has at least three key features: first, it involves a harm or an evil; second, it is imposed and executed on an offender by an appropriate authority (generally the state); and, finally, it is imposed as a response to the commission of a legally prohibited act.^12^ Likewise, it is common to distinguish between two conceptual aspects or dimensions of the harm or evil of legal punishment.^13^ On the one hand, a communicative or symbolic dimension: punishing an offender involves conveying an attitude of disapproval or condemnation (censure) about what the offender has done. On the other hand, a material or afflictive dimension: the punishment also involves the imposition of a burden, a painful or unpleasant consequence on the censured person (hard treatment). What should count as hard treatment, however, remains highly controversial.^14^ The same goes for the interplay between the censure and the hard treatment dimensions of punishment. For the purpose of this article, it is enough to point out that among proponents of an expressionist foundation of punishment, hard treatment is not just a way to make offenders suffer: it is also the appropriate way to convey condemnation or censure.^15^ As John Kleinig puts it: “Hard treatment may register where words fail.”^16^ It is also worth noting that hard treatment would not only make it possible to express condemnation (amplifying function), but it would also make it possible to adjust it (grading function); in other words, the harsher the hard treatment, the greater the censure expressed as a response to the crime.^17^

Of course, advocates of expressivism differ when it comes to making this thesis concrete.^18^ For some scholars, hard treatment establishes essentially a (bilateral) communication relationship between the community and the wrongdoer. For Antony Duff, for instance, the infliction of a burden should allow the latter to focus on their wrongful action in the hope that this will bring about repentance and remorse.^19^ For Christopher Bennett, the hard treatment allows us to communicate how sorry the offender ought to be for the crime committed, that is, how serious we think it is (the degree of our condemnation), by setting a certain level of amends to be made for the crime.^20^ For most scholars, however, punishment operates more as a one-way communicative process. Bill Wringe, for example, argues that the imposition of hard treatment plays a denunciatory role; it is the way in which a punishing authority can express to members of the political community whose laws the offender has broken that the infringement of certain kinds of norms is taken seriously.^21^ Hard treatment, according to Igor Primoratz, operates as vindication of the law.^22^ Tatjana Hörnle, finally, emphasizes the role of the victim: hard treatment is the mechanism through which the reaffirmation of the value of the victim’s rights, which is pursued through punishment, is given serious consideration.^23^

Despite the above (and some other) differences, the expressivist approaches to punishment coincide in two essential aspects. First, the meaning of hard treatment is subjected to a social praxis, i.e., it is conventional. Currently, given the prevailing social conventions in our societies,^24^ it would only be possible to express censure for a criminal offense with sufficient clarity and emphasis through hard treatment.^25^ Thus, it may be theoretically possible at a future time to respond adequately to the most serious crimes without having to resort to hard treatment. While the vehicles that express condemnation are not sacrosanct, for now words alone are not good enough; inflicting pain or withdrawing rights is. Furthermore, social conventions would determine not only the need to resort to hard treatment to accompany relevant acts of censure. Social conventions —and this is the second aspect shared by most expressionist authors— would also determine what counts as hard treatment and what kinds of pain or deprivation of rights are suitable to express the censure that a crime deserves.^26^ Just as champagne is the traditional beverage used in celebrations, imprisonment is currently the paradigmatic way of expressing the reprobation that the commission of a crime deserves.^27^ In Kahan’s words, “liberty is so universally and intensely valued, taking it away is our society’s most potent symbol of moral condemnation.”^28^

This sketch is undoubtedly insufficient in a number of ways, but it serves my purposes for the moment. On the basis of the above premises, some legal philosophers argue that it would currently be impossible to renounce the use of imprisonment as a form of punishment, since the monetary fine (or community service) is unable to express the degree of censure that serious crimes require.^29^ And the problem is not that contemporary systems set an upper limit on the monetary fine that is too low to punish serious crimes. It would be perfectly possible to amend the law to allow for fines of a higher number of daily fees (e.g., fines for life) and of a higher amount (e.g., up to 100% of the offender’s income).^30^ The problem is much deeper: given that monetary fines do not affect innate or very personal interests —such as life, physical integrity, freedom, or honor— but instead affect money —the impersonal instrument of exchange *par excellence*— it would be impossible to express the message of censure in the way that is necessary to respond to (serious) crimes. And note that the problem does not lie in the inability to generate suffering through the deprivation of money^31^; what would be impossible would be to appropriately censure non-minor crimes, given the essentially compensatory connotation that society attributes to the simple transfer of money.^32^

For Peter Young, not only would it be morally wrong not to punish a crime like rape, but it would be morally wrong to do so with a monetary fine, because that would mean putting a price on something constitutive of personhood and individuality. “There exists a core set of values and personal attributes which we resist being brought into contact with money or any other material resource.”^33^ To use the fine to punish a crime like rape would be to convey the message that this kind of harm is compensable, i.e., that everything about a person can be translated into a monetary equivalent. This, however, would be morally repugnant, both to the victim, who would see the perpetrator confronted with a mere commodity like money, and to the society, which would be incapable of highlighting the *proprium* of the harm inherent in a crime like rape. Crime, at least serious crime, for Young, would be included amongst those things that are placed beyond the reach of monetary exchange.^34^ The monetary fine trivializes the seriousness of the offense and denigrates the worth of the victim.^35^ According to Kahan, “when fines are used as a substitute for imprisonment, the message is likely to be that the offenders’ conduct is being priced rather than sanctioned,”^36^ that is, as he also observes, “the offender is being permitted to ‘buy his way out’ of the consequences of his actions.”^37^ He goes on to say that, “while we might believe that charging a high price for a good makes a purchaser suffer, we do not condemn someone for buying what we are willing to sell.”^38^ He argues that the infliction of a pain, i.e., punishment, should give voice to society’s moral outrage.^39^

The above statements ultimately rest on the assumption that imprisonment and the monetary fine are incommensurable —that is, their relative worth cannot be assessed using a common unitary metric^40^— and therefore essentially not interchangeable.^41^ This assumption, in turn, rests on the generally unspoken premise that imprisonment is the penalty *par excellence* for non-minor crimes, the benchmark for all other punishment forms. Any attempt to completely phase out imprisonment would therefore be doomed to failure, as it would be contrary to social conventions that not only determine the need to resort to the infliction of burdens in order to punish, but also require that hard treatment be presented in the form of imprisonment for certain crimes.^42^ There are still some things that, given current social conventions, can be expressed only with walls.^43^

Thus, according to this line of argument, it would be wrong to replace custodial sentences with fines for non-minor crimes. Moreover, according to Duff, the inability to communicate condemnation through monetary fines suggests that we should limit rather than extend their use.^44^ At least for serious crimes, only a prison sentence is an adequate response (the uniqueness claim).^45^ Additionally, it would be correct to insist on conceiving imprisonment as the central punishment for the most serious offenses in a core-periphery penal system model, even though in quantitative terms the fine may be more important.^46^ Kahan argues for the principle of expressive overdetermination, that is, he claims that imprisonment is the kind of punishment that currently raises a greater consensus among different cultural or social groups when it comes to expressing censure.^47^ According to Richard Lippke, “[o]ne plausible account of moral rights is that they designate a group of capabilities whose role is to enable persons to live decent lives shaped by their autonomous choices … [and] serious crimes defeat these capabilities.” His claim is that retributivism requires imprisonment insofar as it, in turn, “significantly diminish[es] the abilities of offenders to live rich and autonomous lives.”^48^ The monetary fine, however, would be an improper and qualitatively different punishment, suitable for sending mere warnings to offenders or punishing offenses such as speeding or health and safety infractions that, in fact, should not have been criminalized.^49^

## The Hard Treatment of Monetary Day-Fines

From now on I assume, first, that punishment does indeed involve both a dimension of censure and a dimension of hard treatment, as well as that the latter operates as the symbolic mechanism through which the censure is conveyed.^50^ Second, I assume a limited conventionalism regarding the ways of expressing censure: how to censure is not something that can be decided individually by those who want to express condemnation, but instead depends on a series of social conventions supported by a social praxis.^51^ The lawmaker, then, is bound hand and foot. (This, of course, does not mean that the legislature is not an agent with the capacity to influence the evolution of our social conventions.) Third, I assume that a criminal offense involves an attack on the individual freedom of others, directly or indirectly, so it is hardly surprising that the mechanism for expressing censure in a stronger and purer way is still to affect the freedom (in a broader sense) of the offender.^52^ All this, however, as I shall now show, does not mean that imprisonment alone offers the degree of censure required to punish serious or eminently personal crimes.

The expressivist’s critique that the monetary fine is inadequate to convey the appropriate condemnation for crime conceives the monetary fine not as an authentic punishment, but instead as a mere monetary obligation, price, or a sort of restitution through which the author is released from their offense.^53^ In fact, as Duff points out, considered externally, the payment of a monetary fine is no different from the payment of a fee, payment for a good or service, or the fulfillment of any other debt to the state.^54^ Thus contemplated, the fine is undoubtedly a symbolically inadequate mechanism for expressing censure. Rather, as Young and Kahan rightly point out, the imposition of a price on the possibility of attacking some of the most important rights (e.g., physical integrity, sexual freedom) expresses and promotes certain attitudes toward these goods that are unacceptable from any imaginable philosophical-political approach. How would a modern state put a price on the possibility of raping another person? However, the approach to the monetary fine as a price or a form of material compensation rests on a normatively incorrect understanding of the fine as a criminal punishment, in particular, of the hard treatment dimension of the monetary fine.

The above-mentioned approaches reduce the afflictive dimension of the monetary fine to the payment of a *quantum* of money, equating the monetary fine to a form of mere restitution for the damage and harm caused by a crime.^55^ This approach also explains the common association between the monetary fine and property and economic crimes.^56^ All this, however, is misconceived. As Georg Simmel pointed out, “Money is the purest form of the tool … it is an institution through which the individual concentrates his activity and possessions in order to attain goals that he could not attain directly.”^57^ It is, in short, the “purest reification of means,” which, through exchange, allows human beings to achieve all the individual goals set for themselves.^58^ Thus, although externally the payment of a fine may be identical to the payment of any other debt, the monetary fine is not simply a matter of the author paying a sum of money as a consequence of the commission of a criminal act.^59^ The payment, in fact, is only the device through which the intended goal of the monetary fine —that is, as in any other form of punishment, the infliction of personal hardship on the offender as a mechanism to reinforce the message of censure associated with the act of public condemnation— is to be achieved.^60^ Obviously, unlike what happens with a custodial sentence, this personal and afflictive effect is not achieved by depriving the convicted person of his freedom of movement; it is done in a different way, but one that is also suitable to convey censure within the framework of contemporary, economically developed, Western capitalist societies: by reducing the consumption ability of the offender for a given period of time as a specific manifestation of the person’s general freedom of action.^61^ Thus, the hard treatment of the monetary fine consists not in the loss of a certain amount of money but in the forced reduction of consumption and thus standard of living (for the duration of the fine installments). The day-fine, in sum, is not a penalty directed toward money, but rather toward what money can buy.^62^

Since the fine does not constitute the mere imposition of a public price or tax on the commission of a crime, nor does it merely make restitution for the harm caused, but rather entails the reduction of the offender’s ability to consume as an essential dimension of his freedom of action,^63^ the monetary day-fine can, in fact, express the censure that characterizes true criminal punishment.^64^ Moreover, given the relevance that the ability to consume has for the vast majority of citizens in contemporary commodity-focused, capitalist societies, it is plausible to affirm that the monetary fine, in accordance with today’s prevailing social conventions, can not only express the censure that misdemeanors deserve, but also serve to adequately punish serious and even eminently personal crimes.^65^ Think, for example, of the impact on the standard of living of a fine reducing the ability to consume to the minimum of subsistence for twenty years. The afflictive effect of such a monetary fine on the condemned person’s ability to carry out his life plans satisfies the normative standard for deciding what counts as an appropriate expressive punishment for serious crimes in the framework of contemporary Western societies. And if that is true, then expressivists might be open to the use of such fines even for serious crimes.^66^

By this I do not mean that all offenses should be effectively punished through monetary fines; perhaps there are certain crimes that are so serious (e.g., murder) that even reducing to the existential minimum the offender’s ability to consume during his or her entire expected life is not enough to express the level of censure that their offense deserves. This, in fact, is not a problem exclusive to the monetary fine: any punishment may, in view of the lifetime of the convicted person, be inadequate to express the required degree of censure. Or perhaps we have good reasons for preferring to deprive some people of their freedom of movement rather than their ability to consume, for example, because we understand that the former resocializes and the latter does not. The only thing I want to argue here is that there are no offenses that, due to their special nature or seriousness, can be adequately censured only through imprisonment.^67^ Or put differently: I cannot see any normative reason why the deprivation of freedom of movement would be more suitable to express the censure that a rape deserves when compared to the deprivation of the ability to consume (with the consequent severe reduction of the offender’s standard of living over a long period of time).^68^

At least one important objection to the account of the hard treatment dimension of monetary fines developed so far might be made. It could be argued that it is an artificial reconstruction of the expressive strength of monetary fines —in short, that I am making a particular use of condemnatory language.^69^ Although the monetary day-fine is legally conceived of as a mechanism to diminish the offender’s ability to consume for a period of time,^70^ this would not be society’s or the victim’s interpretation of the monetary fine. In the eyes of the general public, the fine is seen as a price, and that would be incompatible with the censorious message it is intended to convey. In Kahan’s words, “punishments mean not what a policy advocate would have them mean but what they do in fact mean to the public.”^71^ This criticism highlights the problem that expressionists have in founding and stipulating the social conventions that govern punishment.^72^ I cannot deal with this important topic in depth here. However, it must be stated that assuming the conventionality of the symbols of censure does not mean that they remain in the hands of the simple states of opinion that can be verified at a given moment by survey research.^73^ Rather, the suitability of a form of hard treatment must be assessed with respect to its nature and its compatibility with deep basic social convictions. Thus, assuming that the monetary day-fine is legally configured as a punishment harmful to freedom (deprivation of the liberty to consume) and that the deprivation of liberty in its different dimensions constitutes a mechanism of censure deeply rooted in our society, the repression of (serious) offenses through the day-fine punishment does not imply a radical departure from the wider catalog of —socially stipulated— forms of expression of condemnation.^74^ I can hardly attempt to give a sociological or ethnographic account that elucidates how exactly the monetary fine conveys condemnation in contemporary societies.^75^ In place of such an account, I am content to point out that within a consumer society, the forced reduction of consumption, insofar as it affects a relevant dimension of personal freedom in a manner similar to the rest of the unquestioned censorious penalties, is also a suitable method for expressing censure. That is to say, through the monetary fine, the state does not speak a different language from that of the people; it speaks the same language, although perhaps in a less obvious register —a higher register— than when it resorts to the basic tongue of the prison.^76^

## The Third-Party Problem

From all that has been said so far, it would seem that we should completely replace imprisonment with monetary fines or at least reserve prison as an alternative punishment in the event of fine default. Assuming that serious crimes or crimes against the person can also be punished by fines, it would seem that the ideal punishment in an economically developed society has been found. Unlike imprisonment, the fine would be cheaper for the state and it would not desocialize the convicted person, all the while conveying the message of serious censure for the offense in an appropriate way by drastically reducing the offender’s ability to consume over a long period of time.

Things, however, are not so simple. To assert the appropriateness of the monetary fine as a punishment for serious crimes in the real world, it is not enough to prove that the fine is theoretically suitable to express condemnation. Although largely understudied in the literature, the monetary day-fine presents an essential enforcement problem that ultimately undermines its ability to adequately express censure: the third-party problem.^77^ Since the enforcement of the fine is not based on personal and non-transferable goods —such as life, liberty, physical integrity, or honor— but instead on the extremely fungible and transmissible good that is money, the possibility of shifting the cost of the fine to a (consenting or non-consenting) third party who is not responsible for the crime is a constitutive pathological feature of this kind of punishment.^78^ For one thing, it is possible that a third party may freely decide to bear the cost of the fine, either by paying it directly or by giving the convicted person an equivalent amount of money before or after payment. Think, for example, of the person who —out of pure friendship— pays the fine imposed on an old friend; of the political offender who pays the monetary fine with funds from a “resistance fund” set up by his political supporters; or of the employer who, in order to encourage the efficient breach of the law amongst his employees, regularly pays the monetary fines imposed on them. For another thing, it is possible that the convicted person might disperse the cost of the fine to third parties in his or her social environment.^79^ Consider the offender who diffuses the cost of the monetary fine around his family, for example by reducing his contribution to the shopping basket or by not paying for his daughter’s expensive golf lessons; or consider the convicted person who, in order to pay the monetary fine, proportionally reduces his donations to an NGO that distributes mosquito nets in sub-Saharan Africa.

The scholarship interested in replacing imprisonment with monetary fines as punishment has not yet adequately addressed this problem. My thesis is that the inadequacy of the monetary fine to express condemnation is not explained by the special nature of money, but instead by another reason: there are enormous practical problems that exist in ensuring that monetary day-fines remain personal —that is, that the subject who is censured and the one who suffers the reduction of the ability to consume as a hard treatment are indeed the same person. Although scholars have analyzed the suitability of certain kinds of hard treatment to express censure or condemnation, the relationship of fittingness between the censure judgment and the chosen symbol depends also on the way in which the punishment is enforced.^80^ In other words, the way in which a punishment can be enforced is also crucial in assessing its communicative appropriateness. Just as a custodial sentence in a five-star hotel on the seafront on the island of Mallorca could not express the required censure, a monetary fine that will not necessarily affect the offender can hardly express the censure that a serious crime deserves. It is a pragmatically self-defeating punishment; that is, the way in which the censure message is conveyed to its intended audience undermines the credibility of that message.^81^ Would anyone consider it suitable to express condemnation for a rape by sending the offender’s employer to prison?

Does this mean that the monetary fine is not a proper alternative for punishing serious crimes or crimes against the person? Moreover, should we reconsider the possibility of resorting to fines, given that the dimension of hard treatment does not always fall on the person who is being censured? I believe that both questions should be answered in the negative. However, in order to take the monetary fine seriously, it is essential to ensure that the person whose ability to consume is diminished as a result of the imposition of a fine is the same person who has been found guilty; in other words, it must be ensured that the punishment is a personal one. This, of course, is not a simple problem to solve. The ways in which it is possible to shift the hard treatment of the fine to a third party before or after its enforcement are many and hard to discover and combat. However, I believe that it is possible to minimize this risk to such an extent that it is possible to use fines as a means of expressing censure, even for serious offenses. Here, I must be content to list only the two main strategies of a reform agenda for the enforcement of fines that should be followed to this end.

The first strategy is related to the dispersion of the hard treatment of the fine within the social or family environment of the offender. This is a pathology that is very difficult to control. However, in a criminal justice system that no longer recognizes forms of collective responsibility^82^ and that no longer leaves the enforcement of sentences in the hands of private individuals,^83^ the transfer of the cost of the fine from the convicted person to non-convicted third parties is a major problem. As far as I can see, the only viable legal solution for ameliorating the dispersion of the hard treatment is for the judge to assess the risk of dispersion before determining the kind of penalty and, if there are concrete indications that the monetary fine will not in any way affect the offender’s real consumption ability, to waive the fine in favor of another type of punishment, such as community service.

The second strategy is related to the transfer of hard treatment in cases where third parties freely decide to assume the cost of monetary fines. Few problems arise in the case where a third party intends to directly pay the fine imposed on his or her friend: such payments should not be accepted and, if accepted in error, the monetary fine should be considered unpaid. The problem, of course, is how to prevent convicted persons from paying fines themselves with funds from third parties. Attempts to pay the fines of others (either prior to conviction or after), such that convicted persons do not suffer the intended reduction in their ability to consume, should be criminalized as a form of obstruction of enforcement, in the same way that aiding and abetting someone’s escape from prison is criminalized. And this should apply regardless of whether the convicted person receives the funds before paying the fine or immediately after paying it in full.

Although it is a common thesis among continental criminal law scholars that the transfer of the monetary fine to a third party is not a crime of obstruction of enforcement^84^ as long as the convicted person formally pays the monetary fine themselves, I argue that this conclusion is incompatible with the concept of the monetary fine as a form of punishment. If the hard treatment is the mechanism for expressing censure at the expense of whomever is considered the author of a crime, a correct enforcement of the sentence presupposes that the hard treatment falls on whomever is found guilty —that is, that the punishment affects the censured person.^85^ The enforcement of a sentence need not guarantee that the convicted person will subjectively suffer the consequences of the penalty, but it does need to guarantee that the hard treatment— in our case an objective reduction of the ability to consume for a determined amount of time— will fall on the convicted person whose offense is being censured. In other words, whether convicted persons personally regard the reduction in their ability to consume as a burden or whether they are indifferent to it (for example because, due to the lockdowns accompanying the COVID-19 pandemic, they cannot leave their homes and thus cannot consume) is irrelevant to the proper execution or enforcement of the monetary fine.^86^ What enforcement must guarantee, however, is that the person who is being censured receives the hard treatment dimension of the punishment. Where punishment does not affect the offender, but rather affects a third party, the state has failed in its attempt to punish the offender. The person who uncouples the dimensions of censure and hard treatment of the monetary fine is, thus, obstructing the enforcement and, therefore, committing a wrongdoing analogous to that committed by one who sends another to prison in their place. Ensuring that the monetary fine is also a personal penalty that objectively affects the convicted person is a necessary condition for the fine to express the censure that the commission of a crime deserves.

I will address three objections that might be leveled against my view that monetary fines are a viable alternative to imprisonment. First, scholars might argue that criminalizing the obstruction of punishment enforcement would be unmanageable, insofar as avoiding each and every imaginable maneuver to obscure the origin of the money is infeasible. Consider, for instance, the person who pays with the money from a forty-year-old loan that will never be repaid, or the friend of the convicted who decides to pay his green fee at the golf club every week. Thus, punishing the transfer of the fine would mean punishing only those who are incapable of coming up with minimally sophisticated mechanisms to circumvent justice.^87^ There are, of course, a range of obvious questions that must be addressed concerning how to design judicial control of the appropriate enforcement of the monetary fine. I cannot deal with this problem in depth here. In any case, what I claim is that the risk of not completely eliminating the transfer of the fine to a third party is not a strong argument against punishing the most obvious obstruction maneuvers, but rather an argument in favor of providing judges with effective mechanisms for monitoring the flow of funds between the convicted person and his or her friends and relatives.^88^ The increasing digitalization of such flows should facilitate this task. The existence of a high unrecorded crime rate is not an argument against punishing the crimes that are discovered. Second, some scholars argue that effective control over who ultimately pays the fine would amount to an unreasonable invasion of the privacy of the convicted person and his or her friends and family over a long period of time, even after the execution of the monetary fine has been completed. I recognize that judicial control over the fine that effectively reduces a convicted person’s ability to consume is not an insignificant interference in the privacy of all those involved; however, because I am proposing taking monetary fines seriously in order to make them a viable alternative to prison, I argue that the solution advocated here is preferable from the perspective of privacy rights. Third, scholars might object that, in order to ensure the effectiveness of the fine, a new form of behavior is being criminalized. While it sounds paradoxical, I argue that, in a non-ideal world, ensuring the correct imposition of the monetary fine —even at the cost of imposing community service orders or even custodial sentences against those guilty of the most serious enforcement obstructions— is a fruitful avenue for achieving an overall reduction in the use of custodial sentences.^89^ Only by taking the fine seriously can it be seen as a reasonable alternative to what is still seen as the central punishment of the contemporary penal system.

## Conclusion

Despite the quantitative predominance of the monetary fine in European criminal systems, it has been the subject of very little interest among scholars of sentencing and punishment. Still, expressionist theorists of punishment often agree on two points: first, that a monetary fine is a more humane and efficient punishment than imprisonment and, second, that a monetary fine cannot replace a prison sentence, at least for non-minor offenses. Since a monetary fine consists in nothing more than depriving a person of an amount of money in response to their committing an offense, it cannot express the censure required to punish serious crimes or crimes against the person. A fine would operate as a price or as a compensation mechanism, but it is inappropriate for conveying censure with the clarity and strength required, for instance, to punish a rape.

In this article, I have argued that this conclusion is correct, although not for the reasons usually put forward by expressivists. A monetary punishment is not a price to pay for the crime, nor should it be confused with mere reparation or compensation for the damage caused. The hard treatment of the monetary fine consists rather in reducing offenders’ ability to consume for a certain amount of time, as a fundamental manifestation of their freedom of action. Understood in this way, the fine not only can lead to an a priori severe deprivation of rights (e.g., a fine lasting thirty years that reduces the offender’s standard of living to the minimum of subsistence) but is also in accordance with our actual expressive repertoire for communicating the censure that a serious core crime requires.

I have made the case that the expressive inadequacy of the fine is due to a different problem —one that has been overlooked in the literature on the expressive strength of the monetary fine. Here, I am referring to the (high) risk that the convicted person will shift the hard treatment of the punishment to a (consenting or non-consenting) third party. The risk that fines may be paid by third parties may not be considered relevant when it comes to sanctioning the crimes of corporations, but it does have a fundamental weight in the assessment of the symbolic adequacy of the fine to sanction (serious) crimes committed by natural persons. In this article I have highlighted that, when deciding which symbolic instruments serve to adequately express censure, it is necessary to take into consideration the enforcement of the sentence. Just as a prison without walls, from which everyone could escape, would be unsuitable to convey condemnation, a fine that can be paid by anyone cannot adequately express the censure required.

In any case, the high risk that third parties might pay the monetary fines of others does not mean that we should settle for imprisonment as the only method of punishment appropriate for serious crimes. We cannot be satisfied with a prison sentence as the final state of the process of humanization and rationalization of legal punishment. I have argued that there are ways to sufficiently minimize the transfer of hard treatment to third parties such that the monetary fine can be made capable of expressing the censure that serious crimes deserve. In particular, I posit that it is critical to criminalize as a form of obstruction of punishment any transfer of the monetary fine. Only by taking the enforcement of monetary fines seriously can we make further progress in replacing imprisonment with the criminal monetary fine.

## Footnotes

1 There are, of course, some exceptions. For a discussion of alternative punishments for custodial sentences, see, e.g., Duff (2001, 143–156, 1992).

2 Wilde (2015, 348).

3 For details about the day-fine system, see, e.g., Greene (1988).

4 For the Swedish conception of the day-fines as “temporal fines,” see, e.g., Eriksson/Goodin (2007, 127–128).

5 On their advantages (and limitations), see, e.g., Eriksson/Goodin (2007, 129–131); recently, Schierenbeck (2018, 1876–1884).

6 Obviously, the monetary fine can only work when the convicted person is able to afford it. In states with high poverty and unemployment rates, and without unconditional basic income systems, the fine is not a viable alternative.

7 On the rise of day-fines in the second half of the twentieth century, see Grebing (1978, 28–43).

8 For a good overview see Rusche/Kirchheimer (1968, 166–176).

9 See, e.g., Duff (2001, 146–148); Young (1994), (1989, 64–65); Kahan (1996), (1998a), (1998b). For an introduction to expressivism, see also Hörnle (2017, 31–46).

10 Kahan (1998a, 694) [original emphasis]. According to Husak (2019a, 111), the incommensurability of imprisonment with monetary fines as forms of punishment is the most likely reason that the use of day-fines in the US criminal system remains marginal.

11 Kahan (1999, 51): “That’s the problem for fines and community service: they don’t express condemnation in the same way or to the same degree as imprisonment.” Maybe the death penalty, corporal, and shaming punishments are able to communicate the degree or kind of censure required for punishing serious offenses (or maybe not, see, e.g., Wringe [2017a, 113]), but they show deficient respect for offenders’ dignity and individual rights. For a liberalism-based case against shaming sanctions, see Flanders (2006) and Nussbaum (2006, 227–278). For an argument against the death penalty because of its incompatibility with human dignity, see also Markel (2005).

12 This, of course, is subject to all sorts of refinements. For a general discussion on the definition of (legal) punishment, see, e.g., Nino (1991, 256–261); Boonin (2008, 3–28).

13 Feinberg (1965, 400); von Hirsch (2005, 17); or Silva Sánchez (2018, 113–117).

14 There are basically three points of discussion, albeit closely linked. First, it is controversial whether hard treatment should be understood in a normative way, as deprivation of rights (Nino 1991, 94), a loss (Brooks 2017, 5), a burdensome consequence (Duff 2018, 37), or in a more empirical way, as the infliction of pain or suffering to the offender (Hart [1968, 4]; Newman [1995, 17–27]; Rich [2016, 110–115]). Second, it is discussed whether the notion of hard treatment should be conceived objectively or subjectively; while, for some, it is only relevant that the punishment is the sort of thing that members of society would typically take to be burdensome or painful (Gray 2010; Markel/Flanders 2010), others argue that to punish an offender implies to make them effectively suffer or feel a negative experience (Kolber 2009a, 2009b; Bronsteen/Buccafusco/Masur 2010). Third, it is also discussed whether for an act to be a punishment, it must be done with the intent of harming the person being punished (Boonin [2008, 13–17]; Hanna [2008], 2021) or whether the harm could be simply a foreseen (unintended) side-effect of the punishment (Wringe 2013; Zaibert 2006, 49–58).

15 See, for different versions of this approach, Feinberg (1965); Primoratz (1989); Kleinig (1991); or Hampton (1992). Obviously, there are also expressionist authors like von Hirsch (2005, 21–27) who legitimize hard treatment in a preventive way. For skepticism about expressivism, see Sayre-McCord (2001); Tadros (2011); Hanna (2008).

16 Kleinig (1991, 417), (1998, 275–276).

17 On these two aspects of hard treatment in the expression of penal censure, see recently Matravers (2019, 88–89); von Hirsch (2019, 88–89); further Narayan (1993, 175–182).

18 For the different expressivist approaches to punishment’s audience, see Wringe (2017a, 108–110), (2017b, 682–697).

19 Duff (2001, 107–109); (1986, 236–238).

20 Bennett (2008, 145–146).

21 Wringe (2017a, 106–110), (2016, 18–41).

22 Primoratz (1989, 188, 198).

23 Hörnle (2017, 45).

24 On the concept of conventions, how they are modified, and which ones are relevant to criminal law, see an interesting analysis by Zürcher (2014, 154–158); or Chiao (2019, 255–258).

25 Against the thesis that only hard treatment expresses criticism, see Hanna (2008, 135–148).

26 Zürcher (2014, 153).

27 Feinberg (1965, 402).

28 Kahan (1996, 621), (2005, 2077).

29 Although the discussion on the inability to express censure is not limited to the fine, as this objection is also raised, for example, with regard to community service orders [Kahan (1998a), 701–704; (1996), 625–630], this paper is devoted exclusively to defending the argument that fining satisfies the expressively desirable normative standard for deciding what counts as a proper punishment. I am grateful to an anonymous referee for pressing me to clarify this point.

30 Schierenbeck (2018, 1884).

31 Young (1994, 188).

32 Kahan (1996, 1998a, 1998b, 2005); Young (1994, 1989); Duff (2001, 146–148); Flanders (2006, 613). From a sociological perspective, see also Doob/Marinos (1995, 414–427); Marinos (2005), (1997).

33 Young (1994, 190), (1989, 65).

34 Young (1989, 65).

35 Something different could apply to crimes of material greed. See Duff (2001, 147); (1986, 283–284).

36 Kahan (1996, 621). Similar (1999, 52), (1998a, 697).

37 Kahan (1996, 622).

38 Kahan (2006, 2077).

39 Kahan (1998b, 1641).

40 On punishment incommensurability, see Kahan (1998a, 1996); and Doob/Marinos (1995, 426).

41 For discussion of different substitutability models, see von Hirsch/Wasik/Greene (1989).

42 Duff (2020, 34).

43 Kahan (1998a, 707). Similar Husak (2019b, 37).

44 Duff (1992, 60).

45 A clarification may be helpful here: I am not claiming that all expressivist authors, or even most of them, are staunch supporters of imprisonment. In fact, many of them advocate greater use of non-custodial punishments, in particular community service orders (Duff 2001, 148–152), or giving a more prominent role to restorative justice (Kahan 2006, 2090–2094). What I am asserting is that since these alternatives are at present usually seen as incapable of expressing adequate censure against serious kinds of crime, discarding the monetary fine means practically – even if sometimes reluctantly – assuming imprisonment as the uniquely available proper punishment against those serious crimes. See, e.g., Duff (2001, 148–152). I thank an anonymous referee for alerting me to this potential misunderstanding.

46 Young (1994, 193).

47 On the “expressive overdetermination” of imprisonment, see Kahan (2006, 2085, 2089): “A law or policy can be said to be expressively overdetermined when it bears meanings sufficiently rich in nature and large in number to enable diverse cultural groups to find simultaneously affirmation of their values within it.”.

48 Lippke (2003, 33).

49 For Greco (2018, 200–205) the test for determining whether a behavior deserves to be criminalized is whether it is reasonable to send that person to prison for committing it. In his approach (Roxin/Greco 2020, 23), the fine is only a proper punishment insofar as in case of non-payment, it is substituted by a prison sentence. A similar argument is also developed in von Hirsch (2005, 19).

50 See, e.g., Silva Sánchez (2018, 113–117); Hörnle (1999, 122–124); Kleinig (1991, 417).

51 On punishment conventionalism, see Skillen (1980, 517); Hanna (2008, 130–133); or Zürcher (2014, 153–165).

52 See, e.g., Pawlik (2020, 32–33); or Nussbaum (2006, 238–239).

53 See, e.g., Poama (2015, 116): “[A]rguing that even small fines necessarily or typically generate suffering tends to misrepresent what fining is all about, which is the payment of a sum of money. It is not very plausible to try to describe the payment itself in afflictive terms.”.

54 Duff (2001, 147).

55 Equating fines and restitution to conclude that neither can express censure, see Flanders (2006, 613).

56 See, e.g., Duff (2001, 147).

57 Simmel (1900/2004, 210).

58 Simmel (1900/2004, 210–218).

59 In the same way that the fixing of the amount of a (civil) compensation for a crime such as rape does not involve putting a price on the sexual integrity of the victim, but the approximated restitution for the damage suffered. Young’s (1994, 191–192) argument, however, should logically lead also to the denial of civil restitution for the victim in the case of rape.

60 See, e.g., Baumann (1963, 735); Zipf (1966, 53–55); von Selle (1997, 74–79); or Mühl (2015, 103–108). Similarly, Wringe (2017b, 698): “If a monetary fine is imposed on someone, we expect it to have a negative effect on their well-being.” That the modern monetary fine is not a price but has to “inflict pain upon the culprit” was already pointed out by Simmel (1900/2004, 366).

61 See Zipf (1966, 53–55), or von Selle (1997, 74–79).

62 For a similar approach, see also Morris/Tonry (1990, 129–130, 150), or Eriksson/Goodin (2007, 128–129).

63 On the functional meaning of money as medium of exchange and store of value or wealth, see Proctor (2012, 9–15). For a sociological discussion of the freedom-enabling function of money, see, e.g., Simmel (1900/2004, 210–217), and O’Malley (2009, 72–73), or Faraldo-Cabana (2016, 501).

64 A discussion of the (rough) “interchangeability” of punishments as long as they have equivalent severity can be found in Morris/Tonry (1990, Part I).

65 Rightly so, Baumann (1963).

66 It is not an effective counterargument to simply state that, however long and intense the deprivation of consumption ability, imprisonment will always represent a more severe affliction. The point is not to equate the monetary fine and prison in afflictive terms, but to determine whether the fine can indeed be a normatively adequate response to serious crimes. Where it is, if the hard treatment is not comparable to that of prison, prison should in fact be abandoned. I am grateful to an anonymous referee for suggesting this formulation of my strategy.

67 For a similar view, see Morris/Tonry (1990, 78–79).

68 Obviously, with this understanding of the hard treatment of a monetary fine, I am not solving the problem of how to establish punishments proportionate to an offense through the monetary fine. This is certainly a central problem in any retributive approach to punishment that cannot be solved here. In this regard, see the interesting collective book recently edited by Tonry, in particular the contributions of Duus-Otterström (2020); Husak (2019a); and Matravers (2019).

69 Kahan (1998a, 696) is anticipating this criticism when he affirms the “meanings of different forms of affliction constrain society’s options for constructing punishments.”.

70 There are many legal reasons that show that the monetary day-fine is not a mere compensatory tool. First, it is assessed not according to the damage caused but according to the severity of the offense and the income level of the offender. Second, it is the state that receives the fine, not the victim. Third, it is not executed when the convicted person becomes unable to understand the meaning of punishment (e.g., mental illness) or dies.

71 Kahan (2006, 2087).

72 On the dependence of the type and intensity of punishments on the prevailing culture (mores and sensibilities) of a given society, see, e.g., Garland (1990, Ch. 9); and for a fully “culturalist” approach to the analysis of punishment, see Smith (2008, 169–183).

73 For further discussion see, e.g., Zürcher (2014, 155), for whom the adequacy of hard treatment would not depend on prevailing public opinion or superficial common sense, but on its correspondence to the background of Strawson’s *reactive attitudes* theory.

74 See Galanter/Luban (1993), who make a case for the expressive strength of punitive damages.

75 For further discussion see, e.g., Smith (2008), who, however, does not include the monetary fine in his culturalist study on punishment.

76 Perhaps the cultural or social perception of the censorious force of the monetary fine is not as deeply rooted today as that of imprisonment. However, emphasizing the afflictive force of the monetary fine not only brings to light a penalty preferable to imprisonment but at the same time influences the social convention that stipulates the suitability of deprivation of consumption to express censure. On the conventional meaning-constructing force of praxis and discourse on punishment, see Garland (1990, 213–216). I am indebted to an anonymous referee for pointing this out.

77 This is only a problem for those who, like me, insist on conceiving the fine as a real punishment. Those who, on the contrary, like Barnett (1977), advocate dissolving the boundary between tort and punishment by conceiving the latter as also purely a form of restitution have no problem allowing payment by a third party or even insurance for the criminal fine. For a case against the restitution approach, see Pilon (1978). See also Coca Vila/Pantaleón Díaz (2021), who argue in particular against insuring for criminal fines.

78 This problem is well known among European criminal law scholars. See, e.g., Zipf (1966, 29–32); or recently Faraldo-Cabana (2017, 55–56), 2016, 499).

79 Eriksson/Goodin (2007, 130), Beckett/Harris (2011, 523). To be clear, the dispersion of the hard treatment is not an exclusive problem of the monetary fine, since the execution of custodial sentences and non-custodial sentences (e.g., community service, disqualifications, electronic monitoring) also involves some (collateral) pain or rights restrictions for subjects who are not responsible for the wrongdoing, in particular for relatives and friends of the convicted person. See, e.g., Lippke (2017). However, there is a central difference between this form of dispersal and the one we are interested in here: the dispersal of the hard treatment in those cases does not entail an unburdening of the convicted person but instead additional suffering by third parties – suffering not sought by the state and, moreover, largely beyond the control of the convicted person. Dispersal of the hard treatment of monetary fines, however, not only places an additional burden on the social environment of offenders but also relieves offenders of some or all of the hard treatment of their punishment, and it is precisely offenders who control this dispersal to a large extent.

80 This does not mean that hard treatment cannot be dispensed with in certain cases. In fact, in much of Western Europe, most prison sentences are immediately suspended: only 4 out of every 20 convicted offenders receive unsuspended prison sentences (Aebi et al. 2014). However, the fact that the law contemplates in certain cases the suspension of the execution of the sentence (in the form of parole, for example) is not a convincing argument in favor of allowing a convicted person to freely avoid the hard treatment of the monetary fine at the expense of third parties. Only the latter threatens the stability and legitimacy of the criminal law system.

81 Wringe (2017a, 115); or Pawlik (2020, 33–34): “If the state contents itself with the generous enactment of penal provisions without ensuring their regular enforcement, its performative self-portrayal eventually becomes as untrustworthy as that of the father who rebukes his child for a prank but can hardly resist laughter.”.

82 One might think that to the extent that relatives and friends can benefit directly or indirectly from the crime, it would be fair that they also suffer the punishment drift. This, however, would be to resort to a notion of collective responsibility that is incompatible with the most elementary principles of contemporary liberal penal systems. For further discussion, see Lippke (2017, 656). This is also the reason that monetary fines imposed on corporations cannot be considered genuine criminal punishments. For this second point, see Rich (2016, 114).

83 A state cannot rely on private individuals who initially bear the cost of the fine to subsequently make the convicted person suffer in another equivalent way. For a well-founded case against privately inflicted sanctions, see Harel (2008).

84 Faraldo-Cabana (2017, 64–65).

85 On the relevance of the enforcement in the understanding of the concept and legitimacy of punishment, see, e.g., Rich (2016, 111); or Mañalich (2011, 172).

86 Nor would the prisoner obstruct the enforcement of a custodial sentence if she decided to take a drug that would eliminate any sense of suffering during the time of her imprisonment. To the extent that the prisoner objectively sees her freedom of movement restricted for the time determined in the sentence, the enforcement of the punishment is correct.

87 For more on this, see, e.g., Faraldo-Cabana (2017, 64–65).

88 A potentially fruitful pathway of enforcement would be to authorize judges to deduct the amount of the fine directly from the convicted person’s sources of income – either from his salary or, in the case of an unemployed offender, from his social assistance payments. Obviously, the amount of the fine would always have to be calculated such that a basic minimum for the survival of the convicted person is ensured. I am grateful to a referee for prompting this line of thought.

89 On back-up sanctions for non-custodial penalties, see von (Hirsch/Wasik/Greene 1989, 609–610; Duff 2001, 151–152).
